# Production of eugenol from fungal endophytes *Neopestalotiopsis* sp. and *Diaporthe* sp. isolated from *Cinnamomum loureiroi* leaves

**DOI:** 10.7717/peerj.6427

**Published:** 2019-02-12

**Authors:** Chutima Tanapichatsakul, Sarunpron Khruengsai, Sakon Monggoot, Patcharee Pripdeevech

**Affiliations:** 1 School of Science, Mae Fah Luang University, Muang, Chiang Rai, Thailand; 2 Milott Laboratories Co., Ltd., Bangplee, Samutprakarn, Thailand; 3 Center of Chemical Innovation for Sustainability (CIS), Mae Fah Luang University, Muang, Chiang Rai, Thailand

**Keywords:** Antimicrobial, Eugenol, *Neopestalotiopsis*, *Diaporthe*, *Cinnamomum loureiroi*, Antioxidant

## Abstract

Endophytic fungi, which colonize within a host plant without causing any apparent diseases, have been considered as an important source of bioactive secondary metabolites containing antimicrobial and antioxidant activities. The aim of this research was to isolate the endophytic fungi of *Cinnamomum loureiroi* and then to screen their antimicrobial and antioxidant activities. A total of 11 fungal endophytes were isolated from healthy leaves of *Cinnamomum loureiroi* belonging to six genera: *Botryosphaeria*, *Colletotrichum*, *Diaporthe*, *Fusarium*, *Neopestalotiopsis*, and *Pestalotiopsis*. All isolated strains were cultured and further extracted with ethyl acetate solvent. Antimicrobial activity of all crude endophytic fungal extracts was analyzed using disc diffusion assay against six bacterial and two fungal pathogens. Crude extracts of strains MFLUCC15-1130 and MFLUCC15-1131 showed broad-spectrum antimicrobial activity against all tested pathogens. Activity against *Bacillus cereus* and *Staphylococcus epidermidis* was notable, showing the lowest minimum inhibitory concentration at 3.91 μg/mL. Antioxidant activity of all crude endophytic fungal extracts was also evaluated based on 2,2-diphenyl-1-picrylhydrazyl assay. Significant antioxidant activity was detected in the crude extracts of fungus MFLUCC15-1130 and MFLUCC15-1131 with IC_50_ of 22.92 ± 0.67 and 37.61 ± 0.49 μg/mL, respectively. Using molecular identification, MFLUCC15-1130 and MFLUCC15-1131 were identified as *Neopestalotiopsis* sp. and *Diaporthe* sp., respectively. The major chemical constituents produced by both crude extracts were identified by gas chromatography-mass spectrometry. Eugenol, myristaldehyde, lauric acid, and caprylic acid were the primary antimicrobial and antioxidant compounds in both crude extracts. This is the first report of eugenol being a biologically active compound of *Neopestalotiopsis* sp. and *Diaporthe* sp. fungal endophytes. Eugenol has been reported as antimicrobial and antioxidant agents with agronomic applications. Thus the two newly-isolated endophytes may be used for eugenol production, which in turn can be used in a variety of applications.

## Introduction

Endophytes, mostly identified as fungi and bacteria, are microorganisms residing inside healthy plant hosts without causing any apparent negative effects (Strobel et al., 2004). Most endophytes are classified as ascomycetes and anamorphic fungi (Arnold et al., 2003). Individual plant species may be a host to at least one endophytic fungus, thus the potential of discovering novel endophytic species is high. The relationship between fungal endophytes and plant hosts is intimate. The host plants deliver nutrients to endophytes, whereas endophytes produce secondary metabolites against various pathogenic microorganisms resulting in increasing plant growth rate (Berg, 2009; Ownley, Gwinn & Vega, 2010). Bioactive metabolites produced by fungal endophytes may be applied as sources of novel natural products for exploitation in agriculture, medicine, and other industries (Strobel et al., 2004). Given the broad range of endophytic fungi and their metabolic potential, there are good opportunities to investigate new endophytes colonizing medicinal plants in different ecosystems.

In recent years, the burden of human infectious diseases has increased tremendously worldwide. Current treatment of infections involves using chemical synthetic drugs, which have many side effects. The most notable side effect is development of drug resistance, which has been noted in both human and plant pathogens. Consequently, the effectiveness of old antibiotics is reduced significantly. Thus, there is an urgent need to investigate newer and safer antimicrobial agents. Endophytic fungi may be a better source of antimicrobial compounds than synthetic drugs. Therefore, analysis of antimicrobial activity of natural products produced by endophytic fungi present alternative ways for drug development to control pathogens resistant to antibiotics.

*Cinnamomum* plants belong to the Lauraceae family and contain more than 300 evergreen aromatic plant species (Ravindran, Nirmal-Babu & Shylaja, 2003). They have been also employed as a health promoting agent for the treatment of inflammation, gastrointestinal, and urinary disorders (Brierley & Kelber, 2011) as well as potential antibacterial activity (Al-Jiffri, El-Sayed & Al-Sharif, 2011). Four Cinnamomum species including *Cinnamomum zeylanicum*, *Cinnamomum loureiroi*, *Cinnamomum burmannii*, and *C. aromaticum* are important economic crops because of their numerous culinary uses as spices (Bandara, Uluwaduge & Jansz, 2012). Of these four species, *Cinnamomum loureiroi* has been also used in Chinese traditional medicine as neuroprotective agent (Khasnavis & Pahan, 2012) and for the treatment of diabetes (Kim, Hyun & Choung, 2006). Given the broad range of benefits of *Cinnamomum* plants, some studies have explored the metabolic potential of their endophytic fungi. For instance, *Muscodor albus*, a xylariaceaous fungus isolated from *Cinnamomum zeylanicum*, inhibited fungal and bacterial growth (Strobel et al., 2001). A total of 26 endophytic fungi were isolated from various parts of *Cinnamomum burmannii* and reported to contain antibacterial activity against *Staphylococcus aureus* and *Escherichia coli* (Ilyas, Wulansari & Agusta, 2015). Santiago et al. (2012) reported production of 5-hydroxyramulosin by *Cinnamomum mollissimum*, which inhibited growth of the pathogenic fungus *Aspergillus niger* and murine leukemia cells.

Some endophytes were reported to be able to produce the same set of or similar bioactive compounds as found in their host plants. Production of active compounds by endophytes is a result of a long period of co-evolution between endophytes and their host plants. An example of active compounds produced by endophytes is eugenol, 4-allyl-2-methoxyphenol. It is a volatile phenolic compound presenting in a variety of medicinal plants including plants in the genus Cinnamomum (Chericoni et al., 2005). Eugenol is known to have important pharmacological activities including anaesthetic, antioxidant, antimicrobial, antihelmintic, anti-inflammatory, anticarcinogenic, antifumigant, and antirepellent properties (Bezerra et al., 2017). Although eugenol has been found in many plants, it was also detected in serval endophytes. Cheng et al. (2014) reported that eugenol was isolated from endophytic fungus *Annulohypoxylon stygium* BCRC 34024. Eugenol was also found in bacterial endophytes isolated from *Mentha piperita* reported by Kumar & Mathur (2017). Moreover, methyl eugenol, a eugenol derivative, was also reported to be produced by endophytic fungus in *Alternaria* species from *Rosa damacaena* (Kaul et al., 2008). Production of active compounds by endophytes resulted from long period of co-evolution with a friendly relationship between endophytes and their host plant. Some endophytes were reported to produce the same or similar bioactive compounds as found in their host plants.

Previous studies have found that the endophytic fungi isolated from *Cinnamomum* plants contain several phytochemical constituents inhibiting pathogenic microorganisms. Nonetheless, there has been no study on diversity and chemical profile of endophytic fungi associated with *Cinnamomum loureiroi*. Therefore the purpose of this work was to extract secondary metabolites produced by endophytic fungi isolated from *Cinnamomum loureiroi* and screen for antimicrobial and antioxidant activities. The chemical constituents of fungal endophytes with great antimicrobial and antioxidant activities were also investigated.

## Materials and Methods

### Plant material and chemicals

*Cinnamomum loureiroi* were collected from Mae Fah Luang University Botanical Garden, Mae Fah Luang University, Chaing Rai province, Thailand (20°2′42″N, 99°53′41″E) during May 2015. A voucher herbarium specimen (MFLU No. 10021) was identified and deposited at the Mae Fah Luang University Botanical Garden, Chiang Rai, Thailand. Standard C_11_–C_19_ *n*-alkanes were purchased from Fluka, Germany. Ethyl acetate solvent (analytical reagent grade) was purchased from Merck (Darmstadt, Germany).

### Isolation, culturing, and morphology of fungal endophytes

The surface of healthy *Cinnamomum loureiroi* leaves was pre-sterilized by washing with deionized water. The surface was further sterilized by immersing in 70% ethanol for 60 s, 1% sodium hypochlorite (NaClO) for 60 s prior washing with sterile deionized water for 60 s. The sterilized leaves were cut into 0.5 cm^2^ size and then immediately placed on potato dextrose agar (PDA) medium plate mixed with chloramphenicol antibiotic (100 μg/mL) to suppress the growth of bacteria. The culture medium plates were incubated at room temperature (27 °C) for three days until growth of the fungal hyphae was visible. All endophytic fungi that formed colonies were separated and cultured on new PDA plates and then incubated at room temperature (27 °C) for a week. Seven mycelial agar plugs of endophytic strain were placed into 500 mL Erlenmeyer flask containing 150 mL of potato dextrose broth. The cultures were incubated at room temperature (27 °C) for a month prior to the extracting process. The isolated fungal endophytes were deposited at the Center of Excellence in Fungal Research, Mae Fah Luang University under strain accession MFLUCC15-1112, MFLUCC15-1113, and MFLUCC15-1130–MFLUCC15-1138. The morphological characteristics, for example, colony morphology, growth pattern, hyphae, and spore of isolated fungal endophytes were studied for preliminarily identification.

### Mycelial culture extraction

After incubating for 1 month, the broth culture was filtrated with Whatman™ filter paper No. 3 under vacuum using Buchner funnel to separate the culture broth and mycelia. The medium broth and ground mycelia were portioned and macerated with ethyl acetate with ratio of 1:1 and 1:1.5 v/v, respectively, using modified method of Monggoot et al. (2017). Mycelial fraction was further sonicated using ultrasound-assisted extraction (Crest/690DAE; GuangDong GT Ultrasonic, Guangdong, China) at room temperature for 5 h. All fractions were combined and concentrated by using a rotary evaporator (Buchi Rotavapor Model R114, BUCHI) under reduced pressure. The obtained extract was subsequently stored at 4 °C until further use.

### Screening of antibacterial activity

Antibacterial activity of 11 isolated endophytic fungal extracts was screened against pathogenic bacteria obtained from Department of Medical Sciences, Bangkok, Thailand. Antibacterial activity was evaluated against three Gram-positive (*Bacillus cereus* ATCC 11778, *Staphylococcus aureus* ATCC 25923 and *Staphylococcus epidermidis* ATCC 12228) and three Gram-negative (*E. coli* ATCC 25922, *Pseudomonas aeruginosa* ATCC 9027, *Salmonella typhimurium* ATCC 13311) bacterial pathogens. Antibacterial activities were determined by using a disc diffusion assay. Initially, all crude extracts from isolated fungal endophytes were prepared with a concentration of 1,000 μg/mL using ethyl acetate as a solvent. The obtained solution was then serially diluted using the twofold dilution method until obtaining the concentration of 500, 250, 125, 62.50, 31.25, 7.81, and 3.91 μg/mL, respectively. A single colony of each pathogenic bacterium was activated by culturing in tryptic soy agar medium followed the 0.5 McFarland standard. All pathogenic strains were further cultured on Mueller Hinton agar medium plate. A total of 20 microliters of different diluted solutions were individually loaded into sterilized six mm-diameter filter paper discs (Whatman™, Sigma-Aldrich Corp., St. Louis, MO, USA) and placed onto agar medium plates suppressing bacterial pathogens. Chloramphenicol (20 μg/disc; Sigma-Aldrich, St. Louis, MO, USA) and ethyl acetate solvent was used as positive and negative control, respectively. Plates were then incubated at 37 °C for 24 h. All experiments were performed in triplicate. The inhibition diameter zone values of all endophytic fungal extracts at a concentration of 1,000 μg/mL and chloramphenicol were reported as mean ± standard deviation. The minimum inhibitory concentration (MIC) values were also determined. The variation of inhibition zone values was examined by one-way ANOVA (at *P* < 0.05).

### Screening of antifungal activity

The antifungal activity of all crude endophytic fungal extracts was investigated against *Candida albicans* ATCC 90028 and *Aspergillus niger* ATCC 11414 using disc diffusion assay. All crude extracts were prepared as used in antibacterial activity screening. Both pathogenic fungi were cultured on Sabouraud dextrose agar media and incubated at 30 °C for a week. A plug of 1-week old fungal culture (six mm diameter) was then placed on the center of the sterilized plates and incubated at 30 °C for 5 days. The six mm filter paper discs (no. 3; Whatman, Maidstone, UK) moistened with 20 microliters of each crude extract solution were placed onto the surface of culture plates. The plates were incubated at 30 °C for a week in while the fungal growth was also monitored. The growth inhibition of all endophytic fungal extracts against both fungal strains was measured as the percentage of inhibition of a radical growth relative to the control following formula: Percentage of inhibition (%) = 100 × (1 − (radical growth of treatment (mm)/radical growth of control (mm))). All experiments were performed in triplicate. Ethyl acetate was used as negative control. In addition, MIC value of each sample were evaluated for their antifungal activity against lowest concentration of fungal extract inhibiting the visible growth of each pathogen on the agar plate. The growth inhibition and MIC are shown with the value of mean ± SD.

### Screening of antioxidant activity

The electron or hydrogen atom donation or ability of endophytic fungal extracts was measured from the decolorizing of the purple colored solution of 2,2-diphenyl-1-picrylhydrazyl (DPPH) dissolving in methanol solvent. All endophytic fungal solutions were prepared at various concentrations (1,000, 500, 250, 125, 62.50, 31.25, 7.81, and 3.91 μg/mL) in methanol. One mL of various solutions of fungal extract was added to a one mL of 60 mM DPPH methanolic solution. The solution was mixed vigorously and incubated in dark place at room temperature for 30 min. After incubation time, the absorbance of all solutions was measured at 517 nm with a Perkin–Elmer–Lamda 25 UV/vis spectrophotometer. The antioxidant activity of endophytic fungal extracts was determined as percentage inhibition of DPPH (I%) using following equation: I% = 100 × (Acontrol − Asample)/Acontrol where Acontrol is the absorbance of the control solution (containing all solutions except the fungal extract), and Asample is the absorbance of the crude extract solution. The antioxidant activity of all endophytic fungal extracts was expressed as IC_50_ value (μg/mL) and shown in Table 1. Trolox was used as standard for comparison.

**Table 1:** Antioxidant activity of endophytic fungal extracts and standard drugs, expressed as half maximal inhibitory concentration (IC_50_) values using 2,2-diphenyl-1-picrylhydrazyl radical scavenging assay.

Crude fungal extract/standard	IC_50_ (μg/mL)
*Pestalotiopsis* sp. MFLUCC15-1112	77.23 ± 0.28
*Colletotrichum* sp. MFLUCC15-1113	1,067.25 ± 5.23
*Neopestalotiopsis* sp. MFLUCC15-1130	32.56 ± 0.66
*Diaporthe* sp. MFLUCC15-1131	20.78 ± 0.45
*Botryosphaeria* sp. MFLUCC15-1132	–
*Pestalotiopsis* sp. MFLUCC15-1133	88.36 ± 0.27
*Colletotrichum* sp. MFLUCC15-1134	1,224.23 ± 0.24
*Diaporthe* sp. MFLUCC15-1135	56.32 ± 1.35
*Diaporthe* sp. MFLUCC15-1136	87.14 ± 1.05
*Colletotrichum* sp. MFLUCC15-1137	–
*Fusarium* sp. MFLUCC15-1138	98.04 ± 1.11
Trolox	4.28 ± 0.23
Gallic acid	13.55 ± 0.14

**Note:**

The average IC_50_ value and its standard deviation were calculated from a triplicate experiment. Symbol (–) indicates that the antioxidant activity was not detected.

### Genomic DNA extraction and polymerase chain reaction

MFLUCC15-1130 and MFLUCC15-1131 were selected for genomic DNA extraction and polymerase chain reaction (PCR) due to their notable antibacterial properties. The selected fungal endophytes were grown on PDA for 1 week and the aerial mycelium of each isolate was scraped from the PDA surface. The fungal biomass was then freeze-dried and ground into a fine powder with a pestle and mortar. A modified SDS-CTAB method (Suwannarach et al., 2013) was applied for this experiment. The internal transcribed spacer (ITS) 1-5.8S-ITS2 region of ribosomal DNA was amplified by PCR using ITS4 (5′-TCCTCCGCTTATTGATATGC-3′) and ITS5 (5′-GGAAGTAAAAGTCGTAACAAGG-3′) primers (White et al., 1990) under the following thermal cycling conditions: initial denaturation at 95 °C for 2 min, 30 cycles of 95 °C for 30 s, 50 °C for 30 s 72 °C for 1 min, and final extension at 72 °C for 10 min on a PeqSTAR 2× thermal cycler (Peqlab, Erlangen, Germany). Negative controls lacking fungal DNA were run in parallel. PCR products were checked on 1% agarose gels stained with ethidium bromide under UV light and purified using NucleoSpin® Gel and PCR Clean-up Kit (Macherey-Nagel, Düren, Germany). The purified PCR products were directly sequenced at 1ST Base Company (Malaysia) using the same PCR primers mentioned above. Sequences were used to query GenBank via BLAST (http://blast.ddbj.nig.ac.jp/top-e.html).

### Phylogenetic analysis

The assembled contigs were subjected to BLAST search against the publicly available NCBI database to exclude bacterial contamination and to get a preliminary identification of the genera. Two ITS1-5.8S-ITS2 datasets were assembled. MFLUCC15-1130 and MFLUCC15-1131 were evaluated in a group of *Xylariales* and *Diaporthales*, respectively. Nucleotide sequences were aligned using MAFFT (default parameters) (Katoh & Toh, 2010). The alignment was further subjected to minor manual adjustments and then trimmed with trimAl (automated option) (Capella-Gutiérrez, Silla-Martínez & Gabaldón, 2009). After trimming, MFLUCC15-1130 contained 656 sites while MFLUCC15-1131 had 670 sites. A maximum likelihood phylogenetic tree was constructed using RAxML (Stamatakis, 2006) and the general time reversible model of nucleotide substitution. Bootstrap values were obtained from 1,000 replicates. A Bayesian inference (BI) (Ronquist & Huelsenbeck, 2003) analysis, available on CIPRES Science Gateway (http://www.phylo.org/portal2/home.action) (Miller, Pfeiffer & Schwartz, 2010), was also performed. Four Monte Carlo Markov chains were run for 1,000,000 generations. Convergence was declared when all retention indices (RI) parameters converged toward zero.

### Analysis of volatile compounds by gas chromatography-mass spectrometry

The volatile constituents of crude extracts from strains MFLUCC15-1130 and MFLUCC15-1131 were identified by using a Hewlett Packard model HP6890 gas chromatograph (GC) (Agilent Technologies, Palo Alto, CA, USA), equipped with a HP-5MS (5% phenylpolymethylsiloxane) capillary column (30 m × 0.25 mm × 0.25 μm; Agilent Technologies, Santa Clara, CA, USA). Helium was used as a carrier gas with a flow rate one mL/min, whereas the split ratio of column was operated to 1:50. The oven temperature program was started at 60 °C and increased up to 200 °C, at intervals of 2 °C/min. The GC was coupled to a mass spectrometer (MS) with HP model 5973 (Agilent Technologies, Palo Alto, CA, USA). The electronic ionization energy was set up at 70 eV, while mass range was scanned from 33 to 600 Da with scan rate at 10 spectra/s. Each crude extract was diluted 1:50 in ethyl acetate solvent. Automatically, one μL of solution was injected to the injection port of GC with split mode. The temperature of injector and detector were set at 250 and 280 °C, respectively. Data analysis was performed with ChemStation software (Agilent, Santa Clara, CA, USA). The volatile compounds of crude extract were tentatively identified by comparing the obtained fragmentation patterns of mass spectra with those of verified standards in the National Institute of Standards and Technology database. Linear RI to homologous series of *n*-alkanes, C_8_-C_17_, were calculated to those obtained by Kovat retention index method. Different RI databases of volatile compounds were obtained from Adams (2017), http://www.pherobase.com and http://www.flavornet.org/f_kovats.html.

## Results

### Isolation of fungal endophytes of *Cinnamomum loureiroi* leaves

A total of 11 fungal endophytes were isolated from *Cinnamomum loureiroi* leaves and then deposited at Center of Excellence in Fungal Research, Mae Fah Luang University, Thailand to obtain a strain accession number. Six genera were characterized based on morphological characteristics including conidiophore, growth rate, colony color, and texture. Colonies and morphologies of all isolated endophytic fungi are shown in the Supplemental Material (Fig. S1). *Colletotrichum* genus was detected in stains MFLUCC15-1113, MFLUCC15-1134, and MFLUCC15-1137, *Diaporthe* genus was observed in strains MFLUCC15-1131, MFLUCC15-1135, and MFLUCC15-1136, *Pestalotiopsis* was found in strains MFLUCC15-1112 and MFLUCC15-1133. *Neopestalotiopsis*, *Botryosphaeria*, and *Fusarium* genus was obtained in a single strain from MFLUCC15-1130, MFLUCC15-1132, and MFLUCC15-1138, respectively. The isolated endophytic fungi were cultured in PDA plates with colonies being approximately 6.0–7.0 cm in diameter after incubating at room temperature (27 °C) for a week. Colonies of *Colletotrichum* strain MFLUCC15-1113 were light gray at the border of culture, while the conidial mass of MFLUCC1115-1134 was pale gray at the center and MFLUCC1115-1137 was slightly white and their hyphae were swollen in only half the culture, the pattern of growth layer by *Diaporthe* strains MFLUCC15-1131, MFLUCC15-1135, and MFLUCC15-1136 was grown from mass center. The colony color of MFLUCC15-1131, MFLUCC15-1135 was white, whereas MFLUCC15-1136 presented pastel gray color. *Pestalotiopsis* strain MFLUCC15-1112 was pale gray color with thick-walled and smooth hyphae distribution, while the hyphae of strains MFLUCC15-1130 and MFLUCC15-1133 were septate and formed a white ring colony. Moreover, the sporulation of *Pestalotiopsis* strain was identified by its spore, which consisted of four to five conidia and appendages being dark brown. Colonies of *Fusarium* strains were grayish, but slightly yellow in the center. The growth rate of this genus was rather slow comparing to all other strains in this study. The colony of *Botryosphaeria* strains was white with abundant aerial mycelia.

### Screening of antimicrobial and antioxidant activities

**Figure 1:**
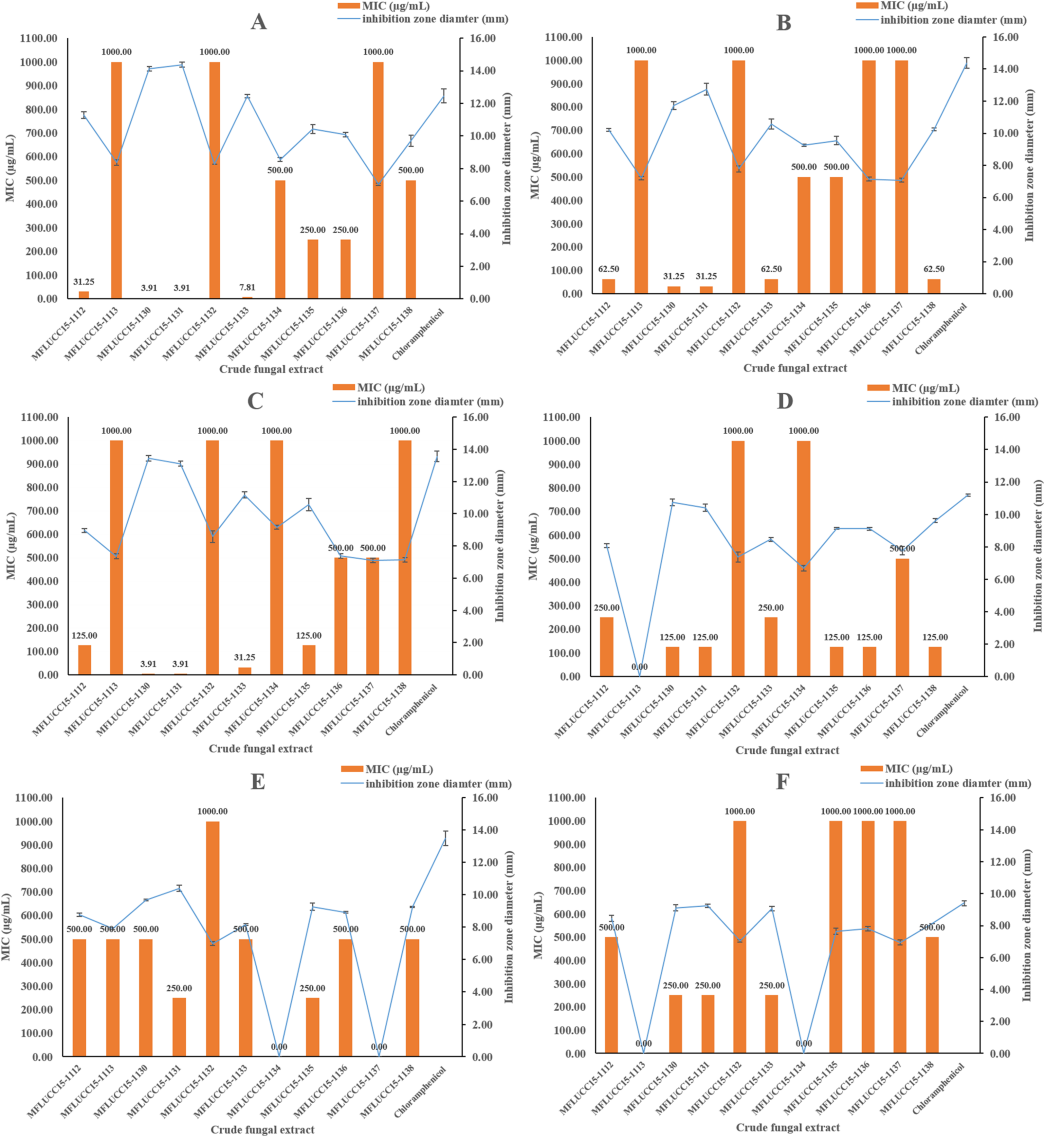
Antibacterial activities of crude fungal extracts and chloramphenicol against *B. cereus* (A), *S. aureus* (B), *S. epidermidis* (C), *E. coli* (D), *P. aeruginosa* (E), and *S. typhimurium* (F), expressed as an inhibition zone diameter and MIC. Each bar graph indicates the average inhibition zone diameter (mm) of crude fungal extracts and chloramphenicol at a concentration of 1,000 μg/mL and 20 μg/mL, respectively (triplicate analysis). No standard deviation value was obtained from this experiment. Each line represents the MIC (μg/mL) of crude fungal extracts against each bacterial pathogen tested. Each error bar was calculated from a standard deviation value derived from a triplicate experiment.

The in vitro antimicrobial activity of 11 endophytic fungal extracts against pathogenic strains from both Gram-positive, Gram-negative bacteria, and fungi was screened using the disc diffusion method by determining inhibition zone diameters. All crude extracts showed antibacterial activities at a concentration of 1,000 μg/mL based on these inhibition zone diameters (Fig. 1). Most fungal extracts were effective on all tested bacteria, with inhibition zones ranging from 7.04 to 14.37 mm. Interestingly, crude extracts of strain MFLUCC15-1130 and MFLUCC15-1131 produced inhibition zone diameter against *B. cereus* much larger than those obtained of chloramphenicol, suggesting that they were more active than this antibiotic. Crude extracts of strains MFLUCC15-1113, MFLUCC15-1132, MFLUCC15-1134, MFLUCC15-1137, and MFLUCC15-1138 presented lower antibacterial activity against test bacterial pathogens than other crude extracts, while broad spectrum antimicrobial activity with the greatest inhibition was observed by crude extracts of fungus MFLUCC15-1130 and MFLUCC15-1131 against all tested pathogens. Crude extracts of fungus MFLUCC15-1113 and MFLUCC15-1132 had no antibacterial activity against *E. coli*, while MFLUCC15-1134 and MFLUCC15-1137 fungal extracts had no effect on *P. aeruginosa*. In addition, MFLUCC15-1113 and MFLUCC15-1134 fungal crude extracts presented less antibacterial activity against *Salmonella typhimurium*. As noticed, Gram-positive bacterial strains were much more sensitive to crude extracts and chloramphenicol than Gram-negative strains. The MIC of antibacterial activity assay was also measured for these 11 endophytic fungal extracts by using the disc diffusion method. The MIC results are shown in Fig. 1. These antibacterial activity assays indicated that crude extracts of strain MFLUCC15-1130 and MFLUCC15-1131 have a very strong activity with MIC ranging from 3.91 to 500 μg/mL and 3.91 to 250 μg/mL, respectively, while the crude extract of strain MFLUCC15-1132 had less antibacterial activity with MIC of 1,000 μg/mL against all tested bacteria. All crude extracts inhibited the growth of Gram-positive bacteria including *B. cereus*, *Staphylococcus aureus*, and *Staphylococcus epidermidis*.

**Figure 2:**
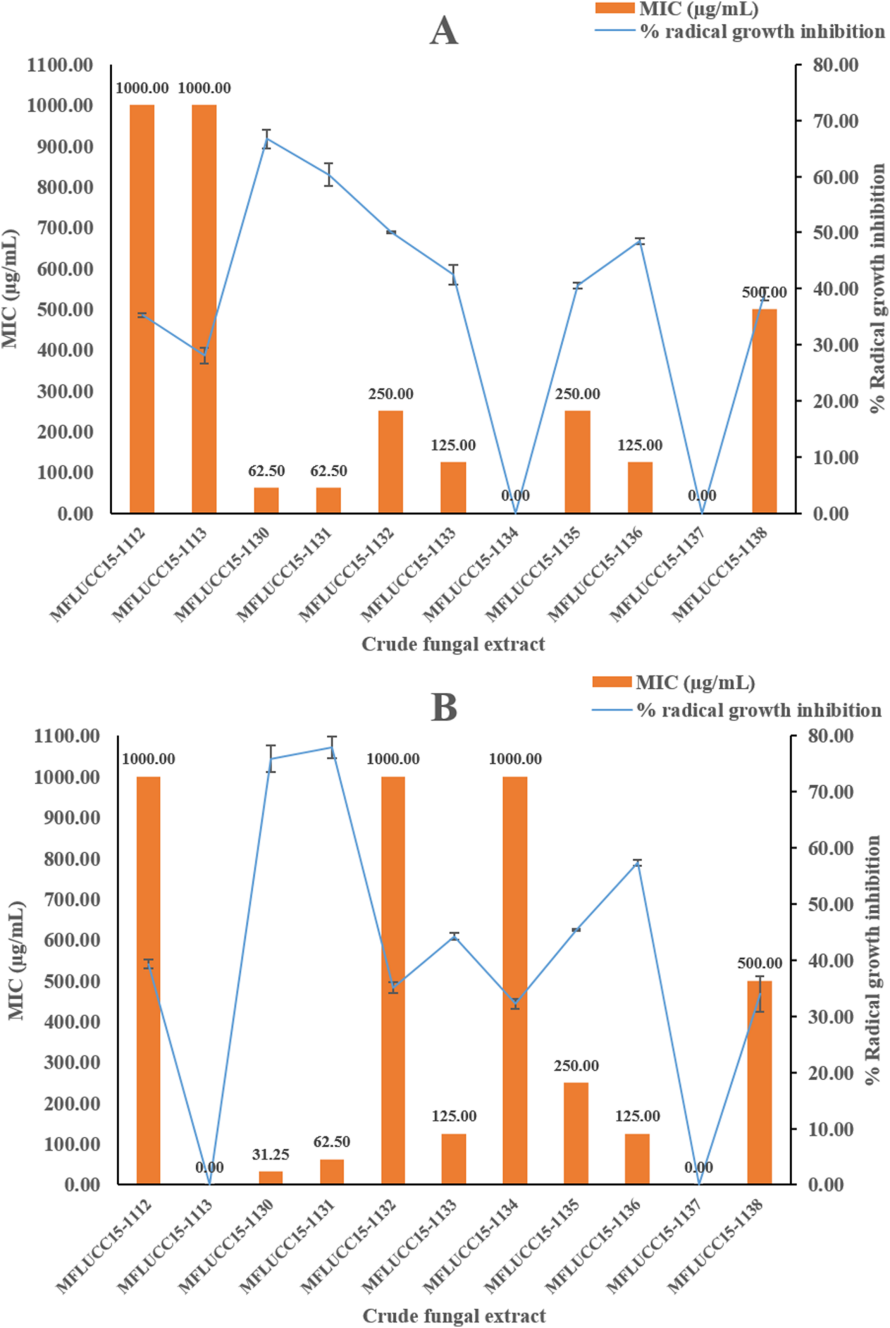
Antifungal activities of crude fungal extracts against *C. albicans* (A) and *A. niger* (B), expressed as percentage of radical growth inhibition and MIC. Each bar graph indicates the percentage of radical growth inhibition of crude fungal extracts at a concentration of 1,000 μg/mL (triplicate analysis). No standard deviation value was obtained from this experiment. Each line indicates the MIC (μg/mL) of crude fungal extracts against each fungal pathogen tested. Each error bar was calculated from a standard deviation value derived from a triplicate experiment.

A disc diffusion assay was also used to investigate the antifungal activity of 11 endophytic fungal extracts at a concentration of 1,000 μg/mL against two fungal strains by determining the percentage of radical growth inhibition. Most crude extracts at concentrations of 1,000 μg/mL showed antifungal activity inhibiting the mycelial growth of *Candida albicans* and *Aspergillus niger* as shown in Fig. 2 ranging from 28.09% to 77.89%. MFLUCC15-1112 and MFLUCC15-1138 crude fungal extracts exhibited a lower inhibition percentage. MFLUCC15-1137 crude extract had no fungicidal activity against *Candida albicans* while no fungicidal activity against *Aspergillus niger* was detected in the crude extracts of fungus MFLUCC15-1113 and MFLUCC15-1134, respectively. The MIC value of all crude extracts against *Candida albicans* and *Aspergillus niger* are also summarized in Fig. 2. The greatest antifungal activity against both tested strains was presented by MFLUCC15-1130 and MFLUCC15-1131 fungal extracts which had MICs of 62.50 and 31.25–62.50 μg/mL, respectively.

The antioxidant activity using DPPH radical scavenging assay was investigated for the 11 fungal extracts. Table 1 shows IC_50_ values of the fungal crude extracts ranging from 22.92 ± 0.67 to >1,000 μg/mL. The crude extracts exhibiting the greatest scavenging effects was obtained from fungus MFLUCC15-1131 with IC_50_ of 22.92 ± 0.67 μg/mL while MFLUCC15-1131 exhibited the second best IC_50_ of 37.61 ± 0.49 μg/mL as compared to the positive control, trolox which was 4.47 ± 0.02 μg/mL, respectively.

### Molecular phylogenetic analysis of MFLUCC15-1130 and MFLUCC15-1131

**Figure 3:**
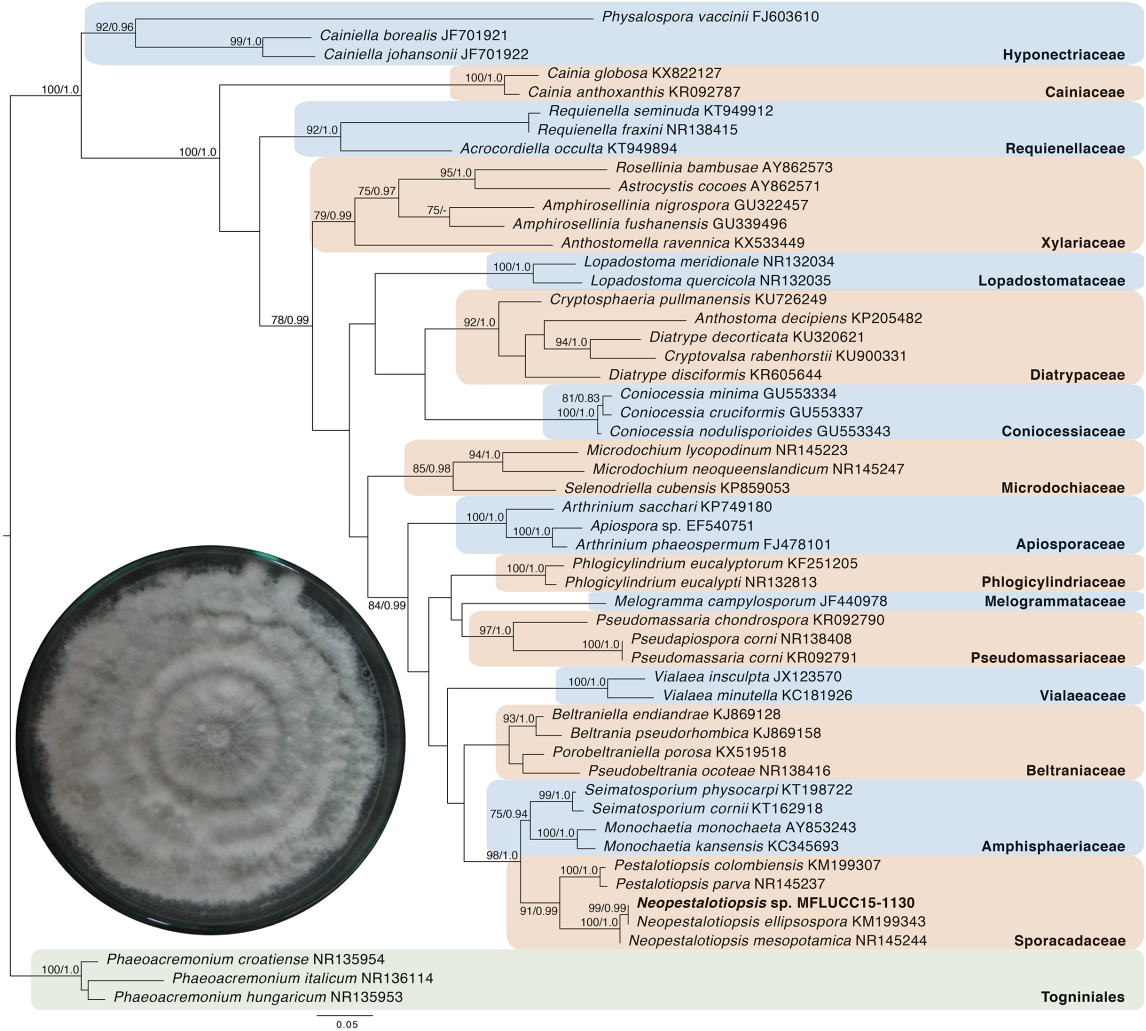
Phylogenic tree of *Neopestalotiopsis* sp. MFLUCC15-1130. This phylogenetic tree was analyzed by ITS4-5.8S-ITS5 inferred 50 taxa under the GTR model of nucleotide substitution +Γ. The newly sequenced species is shown in bold texts. Numerical values at the nodes indicate bootstrap support and posterior probabilities in this order. Only bootstrap support values over 70 are shown.

**Figure 4:**
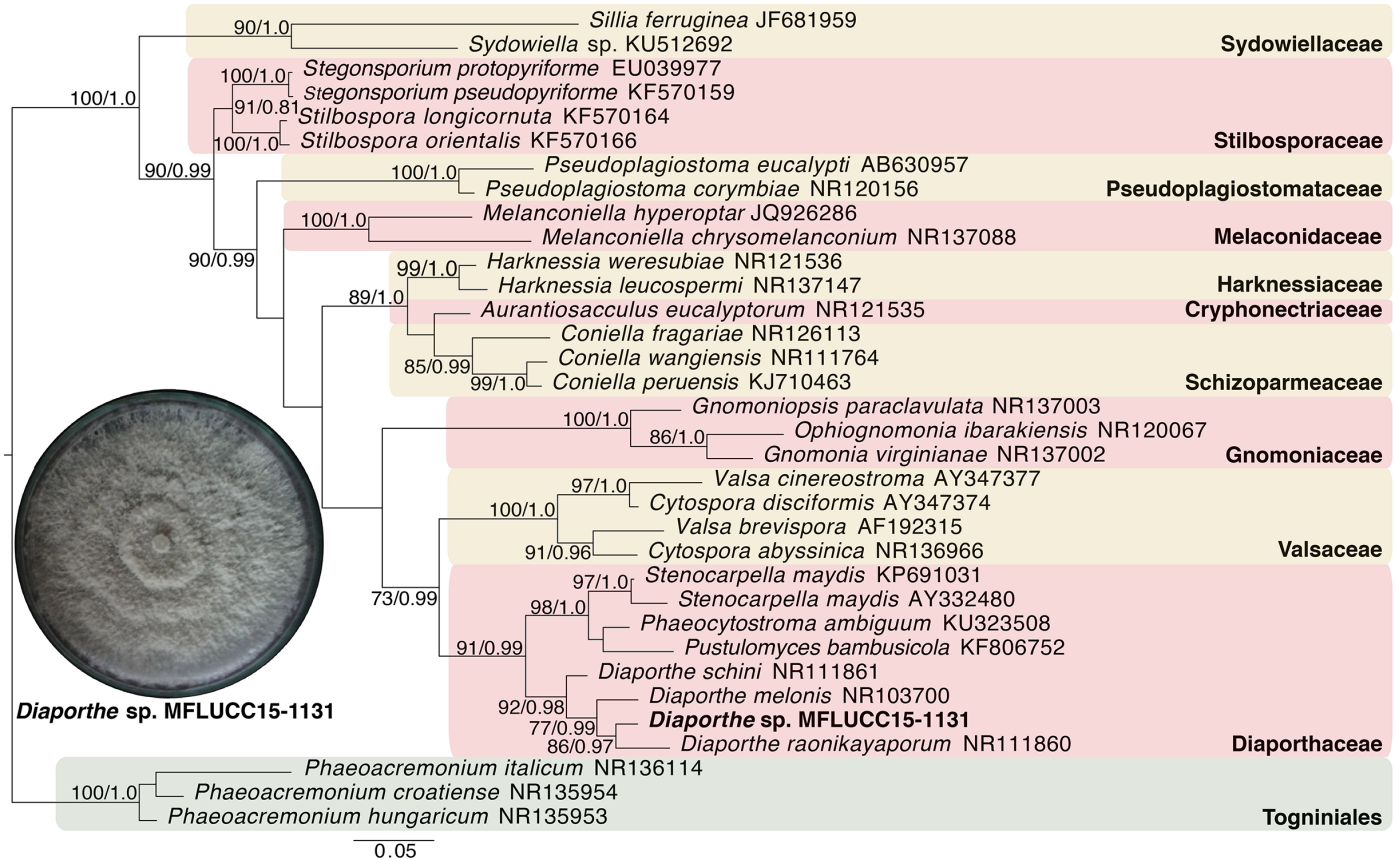
Phylogenic tree of *Diaporthe* sp. MFLUCC15-1131. This phylogenetic tree was analyzed by ITS4-5.8S-ITS5 inferred 31 taxa under the GTR model of nucleotide substitution +Γ. The newly sequenced species is shown in bold texts. Numerical values at the nodes indicate bootstrap support and posterior probabilities in this order. Only bootstrap support values over 70 are shown.

Given the great antimicrobial and antioxidant activities of crude extracts of MFLUCC15-1130 and MFLUCC15-1131, molecular phylogenetic analysis was used to complement morphology. The phylogenetic trees of the endophytic fungal strain MFLUCC15-1130 and MFLUCC15-1131 are shown in Figs. 3 and 4, respectively. Strain MFLUCC15-1130 grouped in the family Sporocadaceae consisting of the genus *Neopestalotiopsis* and *Pestalotiopsis* with a supporting value of 91BS/0.99BI. The new strain sequence was also close to *Neopestalotiopsis ellipsospora* KM199343 with strong supported value of 99BS/0.99BI. Strain sequences of MFLUCC15-1131 grouped in the family Diaporthaceae and within genus *Diaporthe* with a supporting value of 92BS/0.98BI. The new isolate was sister to *Diaporthe raonikayaporum* (86BS/0.97BI). Gene sequences of the *Neopestalotiopsis* sp. MFLUCC15-1130 and strain *Diaporthe* sp. MFLUCC15-1131 were submitted to GenBank database under accession numbers KY646065 and KY646066, respectively.

### Volatile profiles of fungal crude extract of MFLUCC15-1130 and MFLUCC15-1131

The fungal extracts obtained from *Neopestalotiopsis* sp. MFLUCC15-1130 and *Diaporthe* sp. MFLUCC15-1131 were subjected to detailed GC–MS analysis. Identified chemical constituents of crude extracts of fungus *Neopestalotiopsis* sp. MFLUCC15-1130 and *Diaporthe* sp. MFLUCC15-1131 are presented in Table 2. Similar profiles of chemical constituents were obtained among both crude fungal extracts. Exactly 34 constituents were identified in two samples. In *Neopestalotiopsis* sp. MFLUCC15-1130, 34 constituents were identified, representing 97.38% of the total crude extract. Eugenol, lauric acid, myristaldehyde, and caprylic acid were found as the main representatives. A total of 34 constituents were also identified in the *Diaporthe* sp. MFLUCC15-1131, representing 98.70% of the total crude extract. Similarly with *Neopestalotiopsis* sp. MFLUCC15-1130, this sample contained eugenol as the major representative with high content of 59.68%.

**Table 2:** Volatile compounds detected in crude fungal extracts of *Neopestalotiopsis* sp. MFLUCC15-1130 and *Diaporthe* sp. MFLUCC15-1131 using gas chromatography-mass spectrometry (GC–MS).

No.	Compound	RI*	RI**	Relative peak area (%) ± SD	
				*Neopestalotiopsis* sp. MFLUCC15-1130	*Diaporthe* sp. MFLUCC15-1131
1	Isovaleric acid	817	827	3.42 ± 0.07	–
2	Furfural	820	828	0.38 ± 0.30	1.69 ± 0.07
3	2-methyl-Butanoic acid	824	832	0.15 ± 1.18	–
4	4-Pentenoic acid	850	858	0.08 ± 0.71	–
5	2-methyl-Methyl pentanoate	861	871	0.29 ± 0.20	0.15 ± 0.37
6	Methional	888	901	–	3.20 ± 0.02
7	(2E,4E)-Hexadienal	890	907	1.11 ± 0.10	0.62 ± 0.09
8	(2E,4E)-Hexadienol	899	912	0.30 ± 0.19	0.15 ± 0.78
9	Methyl hexanoate	911	921	1.90 ± 0.03	1.12 ± 0.10
10	α-Pinene	930	939	3.19 ± 0.02	1.70 ± 0.07
11	β-Pinene	967	974	–	1.90 ± 0.03
12	3-Octanone	970	979	0.48 ± 0.12	0.20 ± 0.85
13	2-Amylfuran	974	984	0.21 ± 0.27	0.09 ± 0.65
14	Caprylaldehyde	989	998	0.20 ± 0.28	0.09 ± 0.62
15	p-Methyl anisole	1,005	1,015	1.42 ± 0.08	0.71 ± 0.08
16	p-Cymene	1,010	1,020	1.06 ± 0.05	050 ± 0.12
17	o-Cymene	1,012	1,022	–	0.39 ± 0.15
18	trans-2-Octenal	1,035	1,049	0.89 ± 0.06	0.47 ± 0.12
19	5Z-Octenol	1,058	1,065	1.61 ± 0.04	0.73 ± 0.08
20	Caprylic alcohol	1,059	1,063	0.54 ± 0.11	0.39 ± 0.15
21	p-Cresol	1,062	1,071	4.71 ± 0.12	2.50 ± 0.02
22	m-Cymene	1,072	1,082	1.28 ± 0.04	0.68 ± 0.09
23	o-Guaiacol	1,079	1,087	3.20 ± 0.02	1.73 ± 0.33
24	α-Pinene oxide	1,090	1,099	0.75 ± 0.15	0.40 ± 0.41
25	2-Phenylethyl alcohol	1,197	1,106	2.34 ± 0.05	1.23 ± 0.05
26	Octen-3-yl acetate	1,102	1,110	1.97 ± 0.09	1.12 ± 0.10
27	E-Tagetone	1,130	1,139	0.48 ± 0.48	0.38 ± 0.15
28	Z-Tagetone	1,139	1,148	1.09 ± 0.21	1.34 ± 0.04
29	Menthofuran	1,150	1,159	1.88 ± 0.06	1.13 ± 0.05
30	Caprylic acid	1,158	1,167	4.95 ± 0.02	2.58 ± 0.02
31	Methyl chavicol	1,189	1,195	2.57 ± 0.02	1.41 ± 0.04
32	(2E,4Z)-2,4-Decadienal	1,288	1,292	1.41 ± 0.12	0.83 ± 0.07
33	(2E,4E)-2,4-Decadienal	1,310	1,315	2.81 ± 0.04	2.24 ± 0.05
34	Methyl decanoate	1,318	1,323	4.06 ± 0.10	2.36 ± 0.02
35	Eugenol	1,350	1,356	34.12 ± 0.01	59.68 ± 0.01
36	Lauric acid	1,558	1,565	6.79 ± 0.14	2.35 ± 0.05
37	Myristaldehyde	1,604	1,611	5.72 ± 0.31	2.65 ± 0.02
Number of compounds				34	34
Total percentage of relative peak area				97.38	98.70

**Notes:**

For each compound, the average relative peak area and the standard deviation were calculated from a triplicate analysis. Symbol (–) indicates that this compound was not detected in fungal extracts.

RI* values are the self-calculated retention indices on HP-5MS column.

RI** values are the retention indices obtained from the literature using the same column.

## Discussion

In the present work, endophytic fungi of *Cinnamomum loureiroi* leaves were isolated and screened for their antimicrobial and antioxidant activities. *Cinnamomum loureiroi* is known for its antimicrobial activity and other medicinal properties. It is also widely used as food ingredient due to its attractive flavor and as a preservative. Moreover, there are no reports on endophytic fungi from this plant species. A total of 11 fungal endophytes were isolated from host plant in PDA medium plates, which grouped into six genera. Number and type of endophytic fungal genus obtained from *Cinnamomum loureiroi* differed when compared to those found in other *Cinnamomum* plant species. *Muscodor albus* was isolated from *Cinnamomum zeylanicum* and is also a key endophytic fungus of other *Cinnamomum* plants (Strobel et al., 2001). The same genus, *Muscodor cinnamomi* was also found from *Cinnamomum bejolghota* (Suwannarach et al., 2011). This finding implies that a variety of endophytic fungi inhabit different host plant species. The diversity of isolated endophytic fungi may be also largely dependent on the host plant and environment. Exploitation of fungal endophytes in plant tissues provides new possibilities in the search for active compounds. Endophytes are capable of producing novel active compounds that can be used as antimicrobials and antioxidants (Strobel et al., 2004). However, many fungal endophytes and their active compounds remain unknown. Therefore, the endophytic fungi from *Cinnamomum loureiroi* were studied and screened for their antimicrobial and antioxidant potential.

The antimicrobial activity of crude fungal extracts varied possibly due to the mechanism of antimicrobial action of their chemical constituents (Cowan, 1999). In this study, all microbial pathogens showed different sensitivity to the inhibitory action of crude fungal extracts. It was noted that all fungal extracts in this study demonstrated antimicrobial activity on some microbial pathogens, Gram-positive bacteria, Gram-negative bacteria, fungi, or yeast (Gouda et al., 2016). The bacteria and fungi used in this study were selected to cover both human and plant pathogens (Monggoot et al., 2017). The inhibitory activity of all crude fungal extracts indicated their effectiveness against the bacteria and fungi used herein. The crude extracts of *Neopestalotiopsis* sp. MFLUCC15-1130 and *Diaporthe* sp. MFLUCC15-1131 had broad spectrum antimicrobial activity with the strongest antimicrobial activity against six bacterial and two fungal species compared to the other crude fungal extracts. In general, Gram-positive bacterial strains were more sensitive to crude extracts and chloramphenicol than Gram-negative. Because ethyl acetate was chosen as an organic solvent to obtain crude extracts, only organic compounds that have lipophilic/hydrophobic property were able to partition in it. Bacteria that have cell walls comprising with peptidoglycan could easily uptake these compounds into their cells. Diffusion of these organic compounds soluble in ethyl acetate through the microbial membranes results in alterations of membrane fluidity and permeability, disruption of membrane-embedded proteins, inhibition of respiration, and alteration of ion transport processes (Trombetta et al., 2005). The antimicrobial activities of both crude extracts may be attributed to the presence of high concentration of eugenol as reported previously (Devi et al., 2010; Shah, Davidson & Zhong, 2013; Hassan et al., 2018). In addition, the high concentration of other constituents, such as myristaldehyde (Xiangwei et al., 2006), lauric acid (Nakatsuji et al., 2009), caprylic acid (Kim & Rhee, 2016), may be also correlated to the observed antimicrobial activity. Differentiation of chemical constituents may be affected by specific enzyme action on natural biosynthesis pathway (Winter, Behnken & Hertweck, 2011) and different fungal endophyte species. The similarities in the chemical profiles between host and endophyte could stem from the long evolutionary history of symbiosis between host and fungi (Kusari, Hertweck & Hertweck, 2012) and in plant growth association. In addition, some secondary metabolites of the host and its fungal endophytes could have been enhanced with the various nutrients in the media (Dighton, 2003).

The DPPH assay results revealed high antioxidant activity in crude extracts of *Neopestalotiopsis* sp. MFLUCC15-1130 and *Diaporthe* sp. MFLUCC15-1131. High antioxidant activity of both extracts can be explained by the content of eugenol, which was much higher in the case of *Diaporthe* sp. MFLUCC15-1131 in comparison to crude extract of fungus *Neopestalotiopsis* sp. MFLUCC15-1130. It was noted that eugenol was the major antioxidant compounds between the two crude extracts. The antioxidant activity of eugenol, as one of most abundant constituents in investigated crude fungal extracts, has been evaluated using both in vitro and in vivo methods (Ogata et al., 2000; Nagababu et al., 2010; Bezerra et al., 2017). Besides eugenol, synergistic activity of fatty acids, including lauric acid and caprylic acid could also account for the observed differences in antioxidant activity (Mahayothee et al., 2016). High antioxidant activity could also be due to the high amount of non-lipid compounds, such as methyl chavicol (Pripdeevech et al., 2010), p-cymene (De Oliveira et al., 2015) and methional (Sims & Fioriti, 1977), all of which were detected only in *Diaporthe* sp. MFLUCC15-1131.

Recently, eugenol, a major antimicrobial and antioxidant compound detected in *Neopestalotiopsis* sp. MFLUCC15-1130 and *Diaporthe* sp. MFLUCC15-1131, was also detected in various endophytes. Studies by Cheng et al. (2014) and Kumar & Mathur (2017) found that the endophytic fungus *Annulohypoxylon stygium* BCRC 34024, and some bacteria isolated from *Mentha piperita* produced eugenol. Moreover, methyl eugenol, a derivative compound of eugenol was also produced by *Alternaria* species isolated from *Rosa damascaena* as reported by Kaul et al. (2008). This compound has been found as a major constituent in essential oils and extracts of *Cinamomum* host plants (Chericoni et al., 2005; Patel et al., 2007). Both endophytic fungi may have developed the ability to produce similar bioactive compounds as those originating from their host plants. Endophytes from *Neopestalotiopsis* and *Diaporthe* genus were commercially produced bioactive compounds and highly efficient in controlling microbial diseases (Bungihan et al., 2011; Xu, Ebada & Proksch, 2010). Thus, isolation of *Neopestalotiopsis* and *Diaporthe* sp. as endophyte from *Cinnamomum loureiroi* with activity against various pathogens is significant and should be further investigated.

## Conclusions

This study presents a broad spectrum of antimicrobial activities of crude fungal extracts from *Cinnamomum loureiroi* leaves. Antimicrobial efficiency of 11 crude endophytic fungal extracts against six bacterial and two fungal strains was examined. Of the 11 crude fungal extracts studied the ones from *Neopestalotiopsis* sp. MFLUCC15-1130 and *Diaporthe* sp. MFLUCC15-1131 exhibited notable bactericidal and fungicidal activities against all tested bacterial and fungal pathogens in this study. The antioxidant potential of all crude extracts was also assessed for the first time in this plant species. *Neopestalotiopsis* sp. MFLUCC15-1130 and *Diaporthe* sp. MFLUCC15-1131 showed great antioxidant activity. The major constituents found in both endophytic fungi that could responsible for the observed antimicrobial potential were eugenol, lauric acid, myristaldehyde, and caprylic acid. The data reported in this study show that *Neopestalotiopsis* sp. MFLUCC15-1130 and *Diaporthe* sp. MFLUCC15-1131 might provide an alternative safe way to fight microbes and reduce free radical contamination.

## Supplemental Information

10.7717/peerj.6427/supp-1 Supplemental Information 1 Colonies and morphologies of endophytic fungi isolated from *C. loureiroi*. Each colony was 15-day old when the picture was taken. All were cultured at room temperature (27 °C). Their morphology was captured under a microscope (100×).

10.7717/peerj.6427/supp-2 Supplemental Information 2 Raw data of antimicrobial activity. Each sheet represents diameter or MIC of each extract in screening of antimicrobial activity.

10.7717/peerj.6427/supp-3 Supplemental Information 3 Raw data of antioxidant activity. Each sheet presents absorbance at 517 nm and %inhibition including IC50 obtained by DPPH assay.

## Additional Information and Declarations

### DNA Deposition

The following information was supplied regarding the deposition of DNA sequences: Gene sequences of the Neopestalotiopsis sp. MFLUCC15-1130 and strain Diaporthe sp. MFLUCC15-1131 are available in the GenBank database under accession numbers KY646065 and KY646066, respectively.

### Data Availability

The following information was supplied regarding data availability: The raw data of antimicrobial and antioxidant activity are available in the Supplementary Materials.
