# Supplementary figures and images for: Production of eugenol from fungal endophytes Neopestalotiopsis sp. and Diaporthe sp. isolated from Cinnamomum loureiroi leaves
Source: PeerJ. 2019 Feb 12;7:e6427. doi: 10.7717/peerj.6427 (PMC6376936; doi:10.7717/peerj.6427)

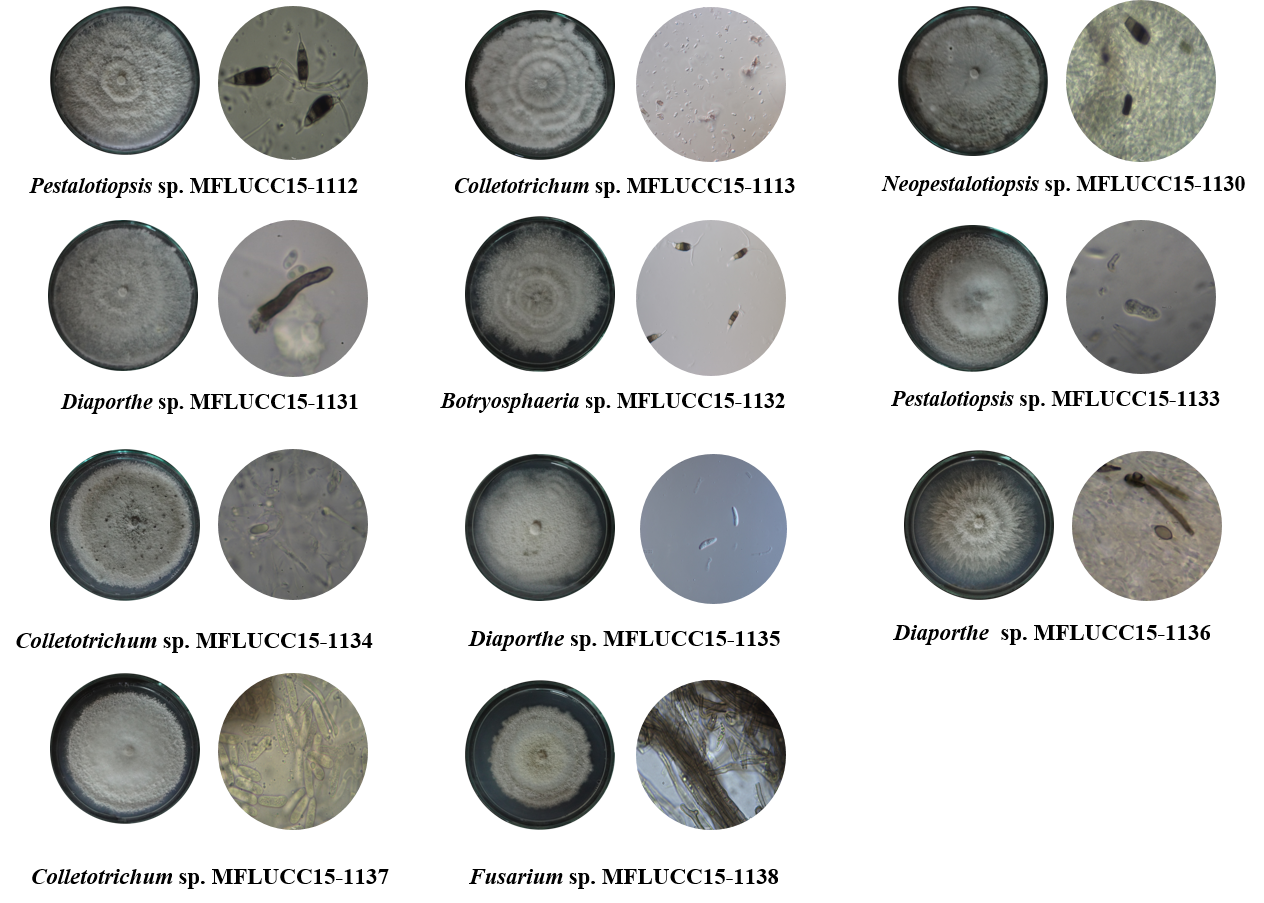

Supplement: Supplemental Information 1 — Each colony was 15-day old when the picture was taken. All were cultured at room temperature (27 °C). Their morphology was captured under a microscope (100×). [file peerj-07-6427-s001.png]
